# Controlling for lesions, kinematics and physiological noise: impact on fMRI results of spastic post-stroke patients

**DOI:** 10.1016/j.mex.2020.101056

**Published:** 2020-09-09

**Authors:** Nabila Brihmat, Kader Boulanouar, Robert Darmana, Arnauld Biganzoli, David Gasq, Evelyne Castel-Lacanal, Philippe Marque, Isabelle Loubinoux

**Affiliations:** a ToNIC, Toulouse NeuroImaging Center, Université de Toulouse, Inserm, UPS, France; b University Hospital of Toulouse, Department of Functional & Physiological Explorations, Toulouse, France; c University Hospital of Toulouse, Department of Rehabilitation and Physical Medicine, Toulouse, France

**Keywords:** Stroke, fMRI, Data processing, Motor task, Lesion masking, Physiological noise, Movement amplitude

## Abstract

Functional magnetic resonance imaging (fMRI) is a widely used technique for assessing brain function in both healthy and pathological populations. Some factors, such as motion, physiological noise and lesion presence, can contribute to signal change and confound the fMRI data, but fMRI data processing techniques have been developed to correct for these confounding effects. Fifteen spastic subacute stroke patients underwent fMRI while performing a highly controlled task (i.e. passive extension of their affected and unaffected wrists). We investigated the impact on activation maps of lesion masking during preprocessing and first- and second-level analyses, and of adding wrist extension amplitudes and physiological data as regressors using the Statistical Parametric Mapping toolbox (SPM12).

We observed a significant decrease in sensorimotor region activation after the addition of lesion masks and movement/physiological regressors during the processing of stroke patients’ fMRI data. Our results demonstrate that:

• The unified segmentation routine results in good normalization accuracy when dealing with stroke lesions regardless of their size;
• Adding a group lesion mask during the second-level analysis seems to be a suitable option when none of the patients have lesions in target regions. Otherwise, no masking is acceptable;
• Movement amplitude is a significant contributor to the sensorimotor activation observed during passive wrist extension in spastic stroke patients;
• Movement features and physiological noise are relevant factors when interpreting for sensorimotor activation in studies of the motor system in patients with brain lesions. They can be added as nuisance covariates during large patient groups’ analyses.

## Graphical abstract

**Figure:**
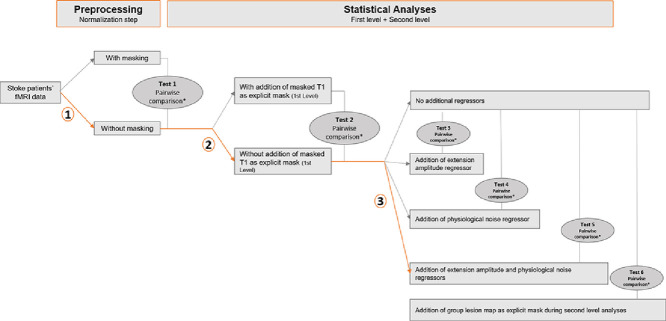

Specifications table

**Table:** 

Subject area:	*Neuroscience*
More specific subject area:	*Neuroscience Methodology* Addressing important issues in the analysis of stroke patients’ fMRI data and refining the processing routine.
Method name:	*Testing the effect of different fMRI data processing strategies using SPM12 toolbox:* • *Effect of lesion masking during fMRI data preprocessing* • *Addition of lesion mask during first-level analysis* • *Addition of group lesion mask during second-level analysis* • *Addition of wrist extension amplitudes and/or physiological noise data as regressors during first-level analysis*
Name and reference of original method:	Lesion masking Brett, M. Spatial Normalization of Brain Images with Focal Lesions Using Cost Function Masking. *Neuroimage***14,** 486–500 (2001). Ashburner, J. & Friston, K. J. Unified segmentation. *Neuroimage***26,** 839–851 (2005). Modelling of cardiovascular noise Kasper, L. *et al.* The PhysIO Toolbox for Modeling Physiological Noise in fMRI Data. *J. Neurosci. Methods***276,** 56–72 (2017). Effect of movement kinematics Casellato, C. *et al.* Simultaneous measurements of kinematics and fMRI: compatibility assessment and case report on recovery evaluation of one stroke patient. *J. Neuroeng. Rehabil.***7,** 49 (2010). Waldvogel, D., van Gelderen, P., Ishii, K. & Hallett, M. The effect of movement amplitude on ctivation in functional magnetic resonance imaging studies. *J. Cereb. Blood Flow Metab.***19,** 1209–1212 (1999).
Resource availability:	MRIcron software (Rorden and Brett, 2000; http://www.sph.sc.edu/comd/rorden/mricron/) *SPM12* (Wellcome Trust Centre for Neuroimaging, London, UK; http://www.fil.ion.ucl.ac.uk/spm/) PhysIO Toolbox (Kasper et al., 2017; http://www.translationalneuromodeling.org/tnu-checkphysretroicor-toolbox/) Labview program (Elliott et al., 2007; http://sine.ni.com/psp/app/doc/p/id/psp-357) OpenSesame Stimulation program (Mathôt et al., 2012; https://osdoc.cogsci.nl/3.2/download/) xjview slice rendering (http://www.alivelearn.net/xjview)

## Method details

### Rationale

fMRI is based on the detection of subtle hemodynamically-driven changes associated with blood oxygenation in tissue and vessels: the so-called blood oxygen level-dependent (BOLD) phenomenon [1,2]. Ideally, there should be no activation other than that resulting from task-specific changes in the BOLD signal in the relevant regions. Unfortunately, numerous other factors may contribute to signal change and confound the fMRI data, such as head motion [3], [4], [5], physiological noise [2,[6], [7], [8], [9], and lesion [10,11] artifacts.

Brain lesions may induce intensity changes owing to inflammation processes, loss or displacement of brain tissues, and dilation of cerebrospinal fluid spaces. This may bias the spatial normalization process and result in sensitivity loss and false negatives. There are several spatial normalization methods that use affine and/or nonlinear warping algorithms and regularize the estimation of warping parameters, including cost function masking (CFM) [10] and the unified automated segmentation technique used in the SPM toolbox [12]. However, best practice for the preprocessing of lesioned brain images is still unclear [13,14].

The analysis of fMRI data obtained from neurological patients affected by movement impairments, such as stroke patients, also requires the monitoring of movement and task compliance [15], [16], [17]. Such movement information is often entered as a regressor in the statistical analysis, to study its impact on brain activation. Frequency-dependent changes in the BOLD signal during finger movements have been studied [4,^18^,19] but there has been little research on the effect of movement amplitude, owing to the need for specific recording material. The predominant hypothesis about movement kinematics is that greater movement amplitude evokes a larger BOLD signal, as supported by Waldvogel et al.’s finding [20]. Other major known sources of signal noise are physiological processes such as heart rate fluctuations, the respiratory cycle [2,21], and the interaction between the two [22], which induce substantial non-neural fluctuations in the BOLD signal. Methods developed to correct for physiological noise are either data-driven or model-based. The latter consider cardiac-related regressor models [6,23] built from data obtained via peripheral recordings of heart rate and/or respiratory cycles. The PhysIO Toolbox [8] is a fully automated model-based physiological noise correction tool that implements various noise models, including RETROICOR. Its flexibility, robustness and capacity for significant noise reduction have been demonstrated in several fMRI studies [8,[24], [25], [26], [27].

Having a clear understanding of the impact of these different strategies on the data and the fMRI motor task-related activation in spastic stroke patients would be useful for deciding which of these strategies to adopt in this particular case.

## Materials

Data were obtained from 15 patients with first-ever stroke resulting in motor impairments (3 females; mean age = 53.7 ± 15.5 years; time since stroke = 5.6 ± 1.7 weeks; mean Fugl-Meyer scale score = 44.8 ± 28.2 /100; mean Tardieu Spasticity Scale score = 2.4 ± 0.8 /4, where 4 indicates severe spasticity). Written consent was obtained from all the patients, in accordance with the Declaration of Helsinki, and the study was approved by the local institutional review board (Comité de Protection des Personnes Sud-Ouest et Outre-Mer II, March 2016).

**Fig. 1:**
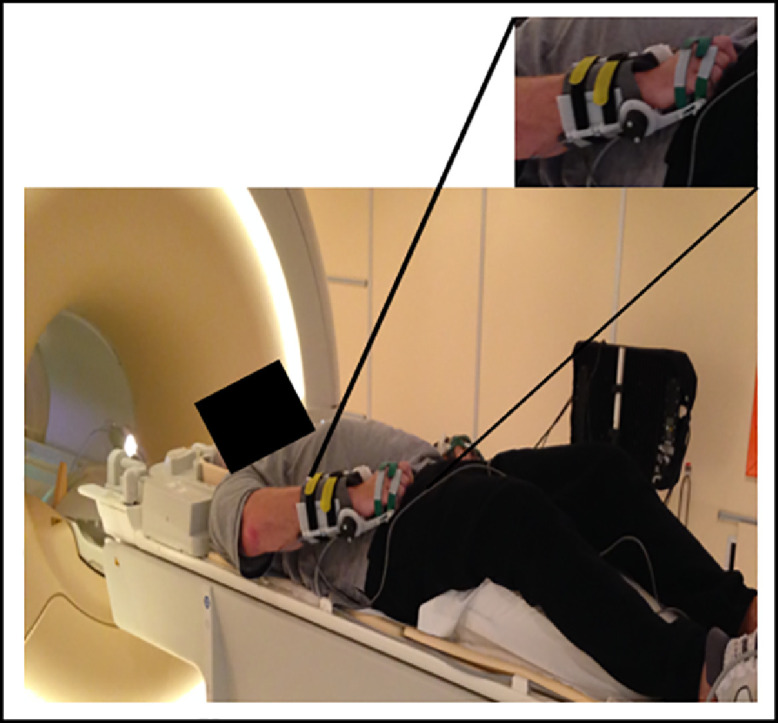
Picture of experimental setup showing homemade MR-compatible goniometer attached to patient's wrist in MRI scanner room. Patient's wrist is at rest, corresponding to an angle of 0°.

During the fMRI examination, patients were in a supine position in the scanner, with their eyes closed. Before each scanning session, they were given instructions and familiarized with the fMRI paradigm. We administered a passive wrist extension task. Task instructions and auditory stimuli indicating movement frequency were provided to the patients through headphones. The auditory stimuli continued across the activation (A) and rest (R) trials of the blocked design paradigm. Each functional run lasted 5 minutes and consisted of 10 30-s trials, alternating between R and A trials. During the R trials, patients were instructed to rest and not to think about the movement, whereas during the A trials, the examiner, who was present in the fMRI room, mobilized the patients’ wrist by raising and lowering their hand from 0° to its maximum amplitude with a frequency of 0.5 Hz. Fifteen passive movements were performed per A trial. Each functional run was performed twice, once using the unaffected wrist and once the affected (i.e. spastic) one. Movement amplitudes were monitored and recorded with homemade MR-compatible goniometers attached to the patients’ wrists (Fig. 1). These goniometers, which did not restrict their movements, were connected to a PC located in the console room. The recording was paced by the MRI trigger. The data were stored digitally, using NI Labview 2009 software (National Instruments Corp., Austin, TX, USA) [28] installed on the PC, and subsequently analyzed offline.

Images were acquired using a Philips dStream Achieva 3T MRI scanner equipped with a 32-channel head coil (Philips Medical Systems, Best, Netherlands). During each of the two functional runs, echo planar imaging (EPI) sequences were acquired (TR = 2500 ms, TE = 3000 ms, flip angle = 90°, FOV = 240 * 240 mm², matrix size = 80*80, voxel size = 3 * 3 * 3 mm^3^, resulting in 47 axial slices per volume parallel to the AC-PC plane). 120 volumes were acquired per run, allowing the acquisition of the entire brain. T1-weighted structural images were acquired using an MPRAGE sequence (TR = 8 ms, TE = 3.7 ms, TI = 520 ms, flip angle = 8°, FOV = 240 * 240 mm², voxel size = 1 * 1 * 1 mm^3^), resulting in the acquisition of 170 sagittal slices. FLAIR images were acquired as part of the routine imaging protocol (TR = 8000 ms, TI = 2400 ms, FOV = 240 * 240 mm², voxel size = 1 * 1 * 1 mm^3^, 170 sagittal slices). The start of the acquisition was synchronized with the auditory stimulus presentation program (i.e. OpenSesame software [29]), and the Labview program was used to monitor and record movement amplitudes during the experiment. Patients’ heartbeat was measured during the functional scans, using an MR-compatible pulse plethysmograph, provided with the MRI scanner, placed on their index finger. The physiological data were sampled at a frequency of 500 Hz, recorded in a scanphyslog file using the scanner software, and were analyzed off line.

## Procedures

### Lesion masking

**Fig. 2:**
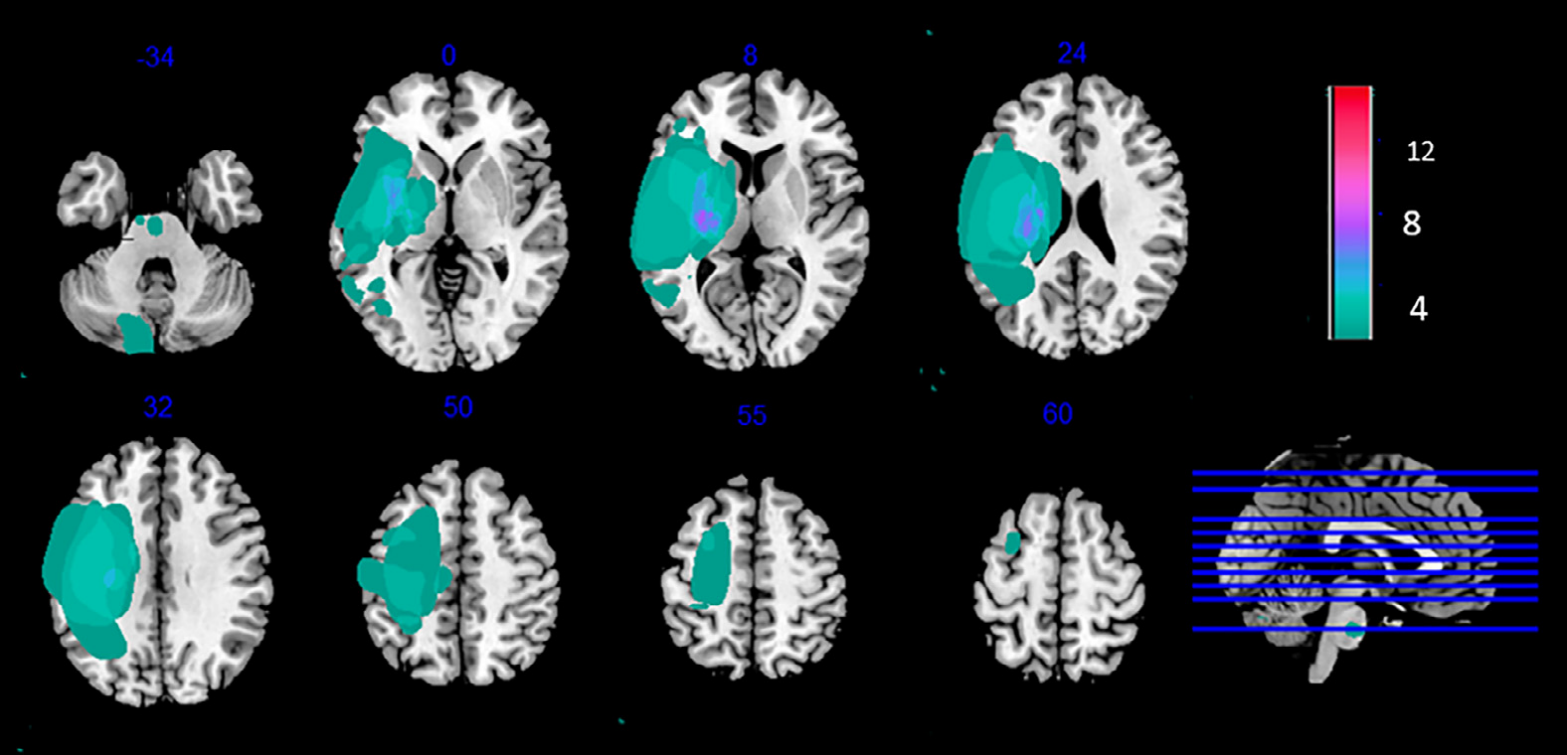
Lesion overlap map of individual lesion masks obtained from spastic stroke patients (*N* = 15). Individual maps were overlaid on a T1 template in MNI space. Right lesions were flipped to left hemisphere. MNI coordinates are given for each transverse section (z-axis), and a sagittal slice is shown for visualization. Color scale indicates number of patients who had a lesion in a given voxel. Stroke lesions were mainly centered on the posterior limb of the internal capsule. Some patients had very large lesions.

For each patient, we created a binary lesion mask depicting the lesion boundaries, using MRIcron software (Rorden and Brett, 2000; http://www.sph.sc.edu/comd/rorden/mricron/). The lesion was first identified using the T1 and FLAIR sequences, after which the lesion volume of interest (VOI) was drawn on each affected slice of the T1 weighted image. The VOI was then smoothed using a 4-mm FWHM Gaussian filter with a 0.1% threshold [10]. A lesion-masked T1 was then created by merging the specific patient's T1 and lesion VOI, using the ‘Imcalc’ function of SPM12 Toolbox (Wellcome Centre for Human Neuroimaging, London, UK; http://www.fil.ion.ucl.ac.uk/spm/) with the formula i1 * .i2. The individual binary lesion masks were normalized and used to create a color-coded lesion overlap map of the lesioned voxels across the brain. Fig. 2 provides an overview of all the patients’ lesioned brain areas.

### Physiological data analysis

The physiological data were analyzed using the physIO Toolbox ([8], http://www.translationalneuromodeling.org/tnu-checkphysretroicor-toolbox/). Cardiac noise modeling took place in five major steps: the reading of the scanphyslog file; synchronization of this file with scan timing parameters; preprocessing of peripheral physiological data; application of a peak detection algorithm to retrieve meaningful physiological measures and improve the signal-to-noise ratio. A third-order cardiac voxelwise phase Fourier expansion was then modelled as a nuisance regressor according to RETROICOR. Thus, the analysis consisted in using the cardiac-related physiological noise regressors we obtained as the “multiple regressors” entry for the first-level generalized linear model (GLM) specification in SPM. The physiological data of three patients were excluded from the analysis, owing to signal loss during the recording and/or poor modeling performance. Only data from the remaining 12 patients were used for the analyses involving physiological regressors.

### fMRI data analysis & results

Preprocessing and fMRI data analysis were carried out using the SPM12 toolbox.

Standard image preprocessing steps were performed. The T1 anatomical volume was segmented (using the unified segmentation model), with or without taking the lesion into account, and normalized. Each patient's functional volumes were corrected for slice timing difference, realigned, co-registered with the T1 anatomical volume, spatially normalized to the Montreal Neurological Institute (MNI) template (using the deformation field calculated during the segmentation step of the T1 anatomical volume), and smoothed using a 6 * 6 * 6 mm^3^ Gaussian kernel, to reduce variability between patients. In order to test for the effect of lesion masking, the preprocessing was performed by taking or not taking each patient's lesion into account during the preprocessing. Quality checks of co-registration, segmentation, and normalization (see Fig. S1) with and without lesion masking revealed no differences.

**Fig. 3:**
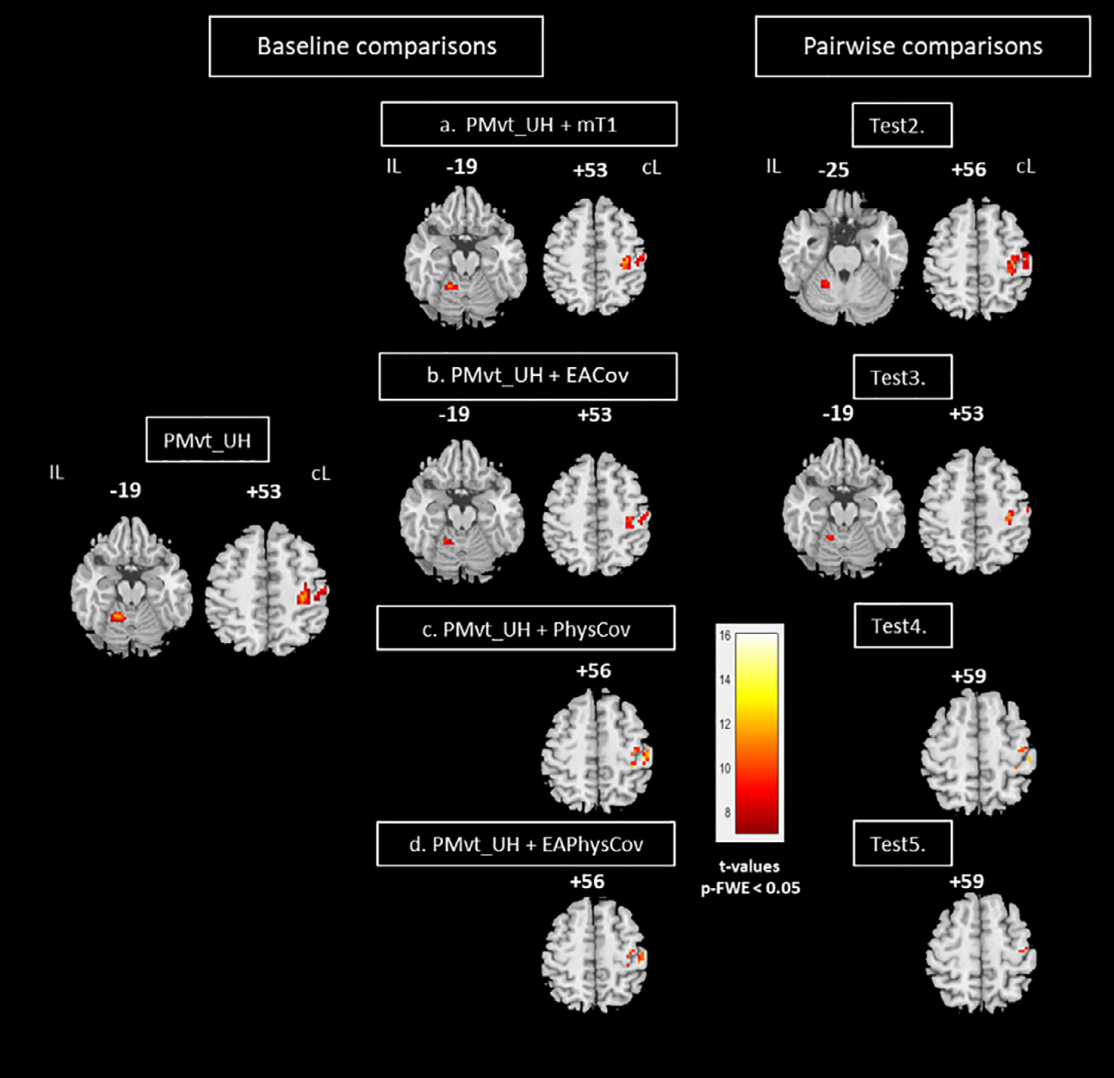
Group comparisons for unaffected hand. Left panel: Brain activation associated with passive extension of unaffected hand (baseline comparison: task vs rest; PMvt_UH). Sensorimotor activation resulting from use of (a) individual lesion-masked T1 as explicit mask during first-level analysis (PMvt_UH + mT1), (b) extension amplitude regressor (PMvt_UH + EACov), (c) cardiac physiological regressor (PMvt_UH + PhysCov), and (d) extension amplitudes and cardiac physiological regressors (PMvt_UH + EAPhysCov) as nuisance covariates. Right panel: Pairwise comparisons between contrast images resulting from different processing pathways (a to d) and baseline condition. Test 2. PMvt_UH > PMvt_UH + mT1. Test 3. PMvt_UH > PMvt_UH + EACov. Test 4. PMvt_UH > PM_UH + PhysCov. Test 5. PMvt_UH > PMvt_UH + EAPhysCov. We found increased activation in the PMvt_UH condition compared with the others, but no decreased activation. Activations are displayed at p < 0.05 familywise error-corrected for multiple comparisons) at the voxel level. Color bars show significance level (t values) for each experimental condition. IL: ipsilesional hemisphere; cL: contralesional hemisphere.

**Fig. 4:**
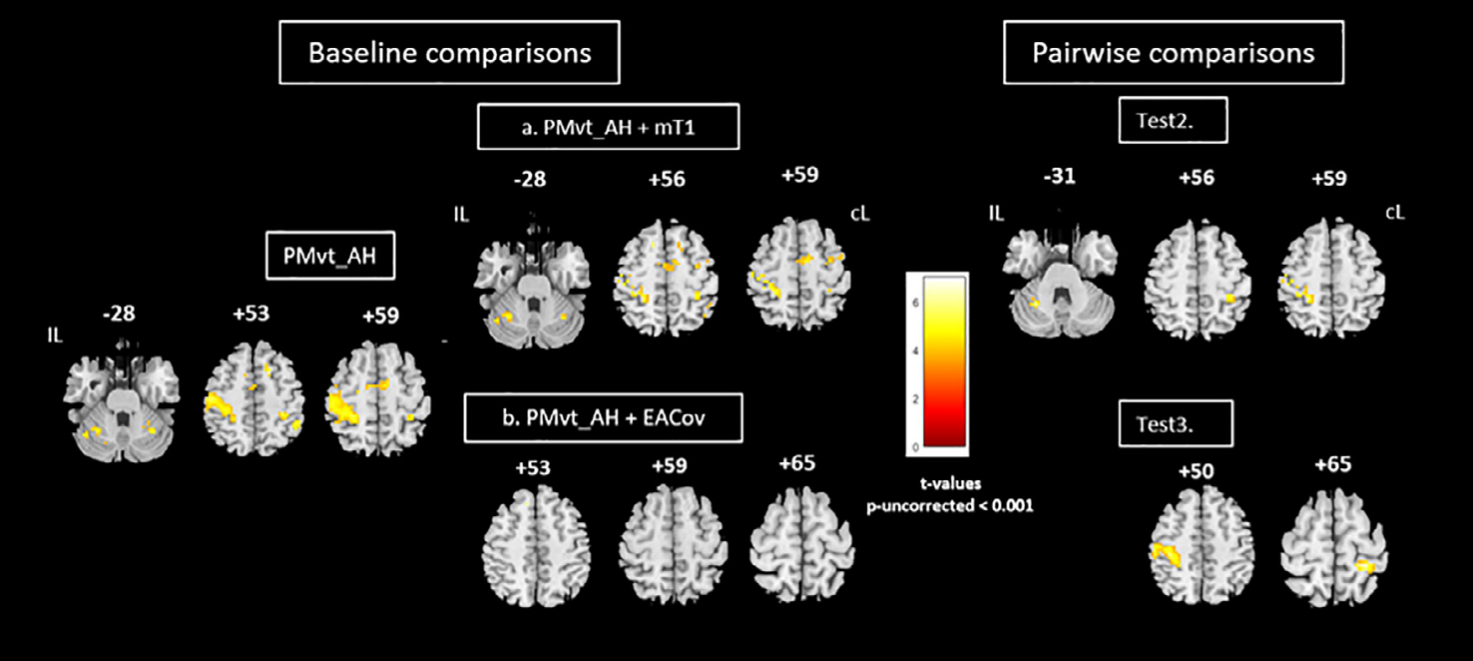
Group comparisons for affected hand. Left panel: Brain activity associated with passive extension of affected wrist (baseline comparison: task vs. rest; PMvt_AH). Brain activation resulting from use of (a) individual lesion-masked T1 as explicit masks during first- level analysis (PMvt_AH + mT1); (b) extension amplitude regressor as covariate (PMvt_AH + EACov). Right panel: Pairwise comparisons between contrast images resulting from different processing pathways (a, b) and baseline condition. Test 2. PMvt_AH > PMvt_AH + mT1. Test 3. PMvt_AH > PMvt_AH + EACov. We found increased activation in the PMvt_AH condition compared with the others, but no decreased activation. Baseline comparisons are displayed at reduced exploratory threshold of p < 0.001 uncorrected, with 40-voxel extent threshold. For pairwise comparisons, small volume correction was applied. Color bars show significance level for each experimental condition (t values). IL: ipsilesional hemisphere; cL: contralesional hemisphere.

For the baseline comparison, individual statistical maps were then computed for each patient, using the GLM implemented in SPM12. Brain areas activated in each of the two experimental tasks-passive movement of the unaffected hand (PMvt_UH; Fig. 3, left panel) and the affected hand (PMvt_AH; Fig. 4, left panel) were revealed with simple effect comparisons (contrast between A and R trials). PMvt_UH significantly activated (familywise error (FWE)-corrected *p* < 0.05) well known sensorimotor areas, including the contralesional sensorimotor cortex (S1M1) and the ipsilesional cerebellum (Fig. 3, left panel). For PMvt_AH, we observed the same pattern of sensorimotor activation, but with less intense and extensive activation in the contralesional S1M1 and ipsilesional cerebellum (Fig. 4, left panel). At a reduced exploratory threshold of *p* < 0.001 uncorrected with a 40-voxel extent threshold, we observed a more bilateral pattern of sensorimotor activation, and the activation of the inferior and medial frontal gyri (ventral and dorsal premotor cortex and supplementary motor area, Brodmann area (BA) 6) and bilateral inferior parietal lobule (BA 40). We found additional activation in the insula (BA 13) and thalamus contralateral to the movement side (Fig. 4). These activation patterns corroborated findings in the literature on stroke patients performing this type of motor task [30], [31], [32].

We then re-ran the first-level analysis for each patient with an explicit mask (patient-specific lesion-masked T1) to exclude the lesioned area from the individual statistical analysis. We also performed analyses with the addition of the normalized wrist extension amplitude and/or physiological noise regressors, to assess their impact on the fMRI results. The single patient contrasts were followed by second-level models. One-sample *t* tests were used to create group maps for each processing pathway and for each experimental task (Figs. 3a-d, and 4a & b). Paired *t* tests were performed between the contrast images resulting from the different processing pathways. Results of the analyses concerning the unaffected hand are displayed at a statistical threshold of *p* < 0.05 FWE-corrected, and Test conditions 3-5 were masked inclusively with the main effect of the PMvt_UH. Given that a previous study among stroke patients had found that activation elicited by the AH was less intense at the sensorimotor region coordinates [31], we searched for effects by applying the small volume corrected method (SVC), using a 15-mm sphere centered on the ipsilesional S1M1, contralesional parietal cortex, and cerebellum coordinates. We applied a corrected threshold of *p* = 0.05. The resulting contrast maps were then overlaid using xjview slice rendering (http://www.alivelearn.net/xjview). The MNI coordinates and the *t* values for each activated area are set out in the supplementary tables.

We then performed six different pairwise tests to look at the effects of each processing strategy:

• *Test 1:* Effect of using individual lesion mask during preprocessing (maskT1N) on activation results (PMvt_UH/AH vs. PMvt_UH/AH + maskT1N) (results not shown)

Pairwise comparisons between the individual functional first-level maps obtained when the lesion was taken into account during the preprocessing of the stroke patients’ fMRI data revealed no significant difference (*p* > 0.05) in group sensorimotor activation, compared with the condition where no masking was applied. Lesion masking during preprocessing may not be responsible for the activation optimization and extension as previously thought [10,14]. CFM seems not to influence normalization accuracy when dealing with images with subacute lesions due to stroke, whatever the lesion size. The resulting segmented and normalized T1 images of one patient with a large lesion (with and without lesion masking) are provided in Supplemental Fig. S1. The unified segmentation routine used in SPM12 seemed to model the lesions better than the other normalization methods (affine or nonlinear transformations), even in the case of large lesions (Fig. S1). The CFM procedure does not really exclude the area from the process, but rather implements the solution applied to the unmasked portions of the image to the area under the mask [11]. It improves normalization and provides more sensitive results. However, even if drawing precision is not a critical parameter to be taken into account [11,15], the actual manual masking procedure is time consuming, particularly in the case of large cortical-subcortical lesions that affect almost all the slices. Furthermore, besides its subjectivity [12,34], there may be other abnormalities in the lesioned brain outside those drawn within the mask, meaning that the normalization process is still not optimal. We therefore suggest that lesion masking may not be a necessary step during preprocessing in cases where the unified segmentation routine is used [14,35]. We therefore decided to use images that had been preprocessed without lesion masking for the subsequent analyses (Path 1).

• *Test 2:* Effect of adding masked-T1 as explicit mask during first-level analysis (PMvt_UH/AH vs. PMvt_UH/AH + mT1)

Explicitly masking the individual first-level statistical analysis with a lesion-masked T1 significantly reduced the level of activation of the contralesional S1M1 and ipsilesional cerebellum (*p* -FWE-corrected < 0.05) induced by the passive unaffected wrist extension task in the PMvt_UH > PMvt_UH + mT1 contrast (Fig. 3, Test 2). We found a similar result (*p* < 0.05 SVC) for the affected hand (Fig. 4, *Test 2*).

The individual masks were drawn on high-resolution T1 images, and the lesions may have been slightly overestimated when applied on less resolved EPI images during the first-level analyses, resulting in the loss of perilesional activation elicited by the passive wrist movement that may have reflected reorganization processes. Moreover, the excluded voxels were not only in the perilesional areas. Distant voxels were also excluded from the analysis after the addition of the masks. Given these results, and to avoid carryover effects of the different strategies on the subsequent analyses, we decided not to explicitly mask at the first level (Path 2).

• *Test 3:* Effect of adding *extension amplitude regressors* as covariates (PMvt_UH/AH vs. PMvt_UH/AH + EACov)

The S1M1 and cerebellar activations observed during PMvt_UH (Fig. 3) and PMvt_AH (Fig. 4) were significantly decreased by the addition of extension amplitudes as regressors during the first-level analysis (Figs. 3 and 4, right panels). There was no difference in cerebellar activation for the affected hand (Fig. 4, *Test 3*). Regression analysis on the group revealed a positive correlation between patients’ mean and maximum wrist extension amplitudes and the S1M1 and cerebellum activation observed during PMvt_UH, and S1M1 activation during PMvt_AH (*p* < 0.05; results not shown).

Movement features and their associated cortical activations can enrich fMRI obtained information and make the underlying results easier to interpret [16,^20^,^33^,34]. In the study by Caselatto et al. [16], the addition of kinematic regressors did not add any new information about a healthy participant, and the resulting cortical maps remained unchanged. However, in the case of a hemiparetic patient, it optimized the activation map, by extending it. This result was not observed in our group of spastic stroke patients with heterogeneous lesion locations and sizes, probably because the movement was passive, and perfectly timed and controlled. Differences in movement kinematics may have been due either to intra-individual lowering of wrist extension amplitudes resulting from spasticity triggered by movement in the course of the experiment, or to increased compensatory muscle contractions. They may have led to the positive correlation observed, responsible for the reduced motor activation when the amplitude regressor was included. These parameters seemed to contribute significantly to sensorimotor activation during PMvt_AH. This shows the need to control and report movement kinematics such as rate and amplitude during fMRI experiments, especially when dealing with voluntary movements, which are less easy to control. This conclusion is even more relevant when dealing with an impaired population where not all the patients have the same kinematics for a given movement. Quantifying movement kinematics and including them during model estimation may be crucial for interpreting the fMRI results, in order to link the observed differences to differences in movement parameters, occurrence of involuntary movements, effect of rehabilitation intervention, or recovery [15,^16^,19]. Once this has been done, some authors may choose to keep or not the movement amplitude variable, depending on their recording conditions and purpose.

• *Test 4:* Effect of adding cardiac physiological regressors as nuisance covariates (PMvt_UH/AH vs. PMvt_UH/AH + PhysCov)

**Fig. 5:**
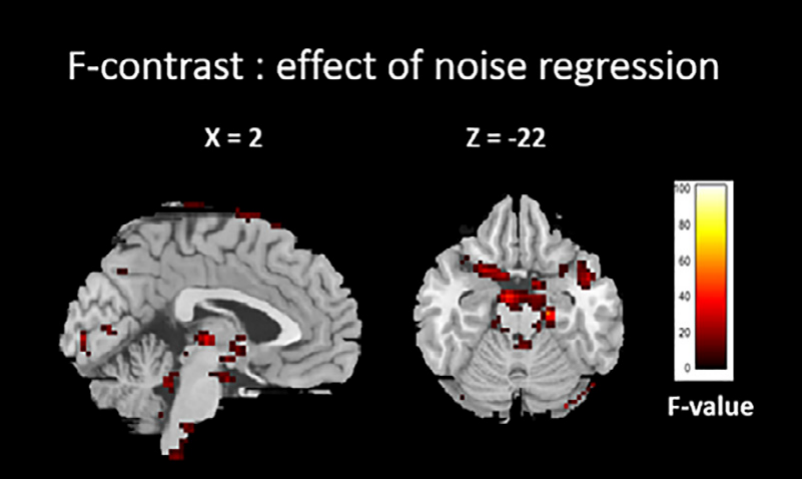
F-contrast showing result of adding cardiac physiological regressor as nuisance covariate in first-level analysis.

We performed an F-contrast in order to look at the effect of adding the cardiac physiological regressors as nuisance covariates during the first-level analysis and thus to confirm its relevance. The F-map for one patient is shown in Fig. 5. The brainstem, pons, cerebellar boundaries, and ventricles match the known cardiac noise sites reported in the literature [8,[35], [36], [37]. This pattern was observed for most patients, thus suggesting that the PhysIO Toolbox offers effective model-based cardiac noise correction.

Adding cardiac physiological regressors as nuisance covariates also significantly reduced the sensorimotor activation seen during PMvt_UH (Fig. 3, *Test 4*). For PMvt_AH, no significant differences were found, even when the SVC was applied.

Many studies have discussed the impact of physiological noise on the sensitivity of fMRI analyses. An increase in heart rate and respiratory variation is observed during active periods of task-related paradigms, resulting in a correlation between the nuisance regressors and the fMRI contrast [38]. These task-correlated physiological fluctuations result in decreased specificity of the fMRI signal regarding the true neural signal [35]. Inclusion of nuisance regressors for cardiac and respiratory fluctuations can thus remove the bias in the contrast estimates. The PhysIO Toolbox is especially to be recommended, given its robustness and ease of use, particularly when dealing with pathological populations more prone to motion and physiological artifacts [39]. The quality of the results does, however, have to be checked, given that in some cases, the patient's heartbeat may not be detected, owing to say excessive motion or signal loss, and may thus result in poor physiological noise modeling and correction. It may also be advisable to model respiratory fluctuations.

• *Test 5:* Effect of adding extension amplitude and cardiac physiological regressors as covariates (PMvt_UH/AH vs. PMvt_UH/AH + EAPhysCov)

Adding both extension amplitude and cardiac physiological regressors as covariates significantly decreased the sensorimotor activation resulting from PMvt_UH (Fig. 4, *Test 5*). No differences were found for cerebellar activity when analyzing the activation resulting from PMvt_AH. The absence of an effect for the affected hand can be explained by the small number of patients included in this analysis (12 out of 15) and the lower residual activation of the lesioned hemisphere, which made it harder to find significant results. Another hypothesis is that the movement was more difficult to perform with the affected wrist, given that some patients may have experienced spasticity during the task trials, limiting completion of the task and thus its physiological correlates.

However, even if this last strategy was responsible for a loss of activation, applying physiological noise correction and considering movement kinematics information may make it easier to interpret the results and draw a more direct link between the brain activation that is observed and the task performed by the studied population. We therefore recommend the final path (Path 3) before the subsequent second-level analysis.

• *Test 6:* Effect of adding *a* group lesion map as an explicit mask during the second-level analysis

Interestingly, the addition of the group lesion map (Fig. 2) as an explicit mask during the second-level analysis did not significantly affect the degree of activation (result not shown). The maps resulting from the pairwise comparisons with nonmasked baseline contrasts seems to have only removed a few voxels in the sensorimotor regions. The problem of excluded distant voxels raised in *Test 2* did not arise when we used the group lesion mask during the second-level analysis. Thus, masking at the group level may be more appropriate when none of the patients have lesions in the target regions. Otherwise, no masking is acceptable. In our study, we deemed that sensorimotor activation was relevant for the patients group, thus we chose to not mask at the second-level.

## Final considerations

In the present study, we explored the effects of different processing strategies on the fMRI results of a selected portion of the stroke population, namely spastic stroke patients. The latter have more severe motor impairments, and their spasticity hinders their ability to perform motor tasks and the interpretation of the results. Even though some processing strategies (i.e. addition of kinematic and physiological data as regressors) seem to reduce the degree of cerebral activation observed, they also help to identify key factors and make the observed results easier to interpret. A direct link can thus be drawn between the activation observed and task-specific changes in the BOLD signal in the relevant regions. Consequently, in similar conditions, we recommend the three paths highlighted in the graphical abstract to analyze spastic stroke fMRI data. Of course, this is even more suitable when dealing with large groups of stroke patients with strong task-related BOLD signals. There is now a need to standardize the neuroimaging processing method. The differences that are sometimes observed between similarly designed studies may partly be explained by differences in processing methods [[40], [41]]. One solution would be to design standardized procedures, validated by a panel of neuroimaging experts, which could then be integrated into scanners’ processing software. The choice of these procedures would depend on the study design and the population being studied. This would yield preprocessed images and results that would allow for easier comparisons between neuroimaging studies.

## Declaration of Competing Interest

The Authors declare that there is no conflict of interest.

## Appendix Supplementary materials

Image, application 1
