# Supplementary material for: Controlling for lesions, kinematics and physiological noise: impact on fMRI results of spastic post-stroke patients
Source: MethodsX. 2020 Sep 9;7:101056. doi: 10.1016/j.mex.2020.101056 (PMC7509233; doi:10.1016/j.mex.2020.101056)
Supplement: Supplementary file 1 [file mmc1.docx]

**Supplementary material *and/or* Additional information**


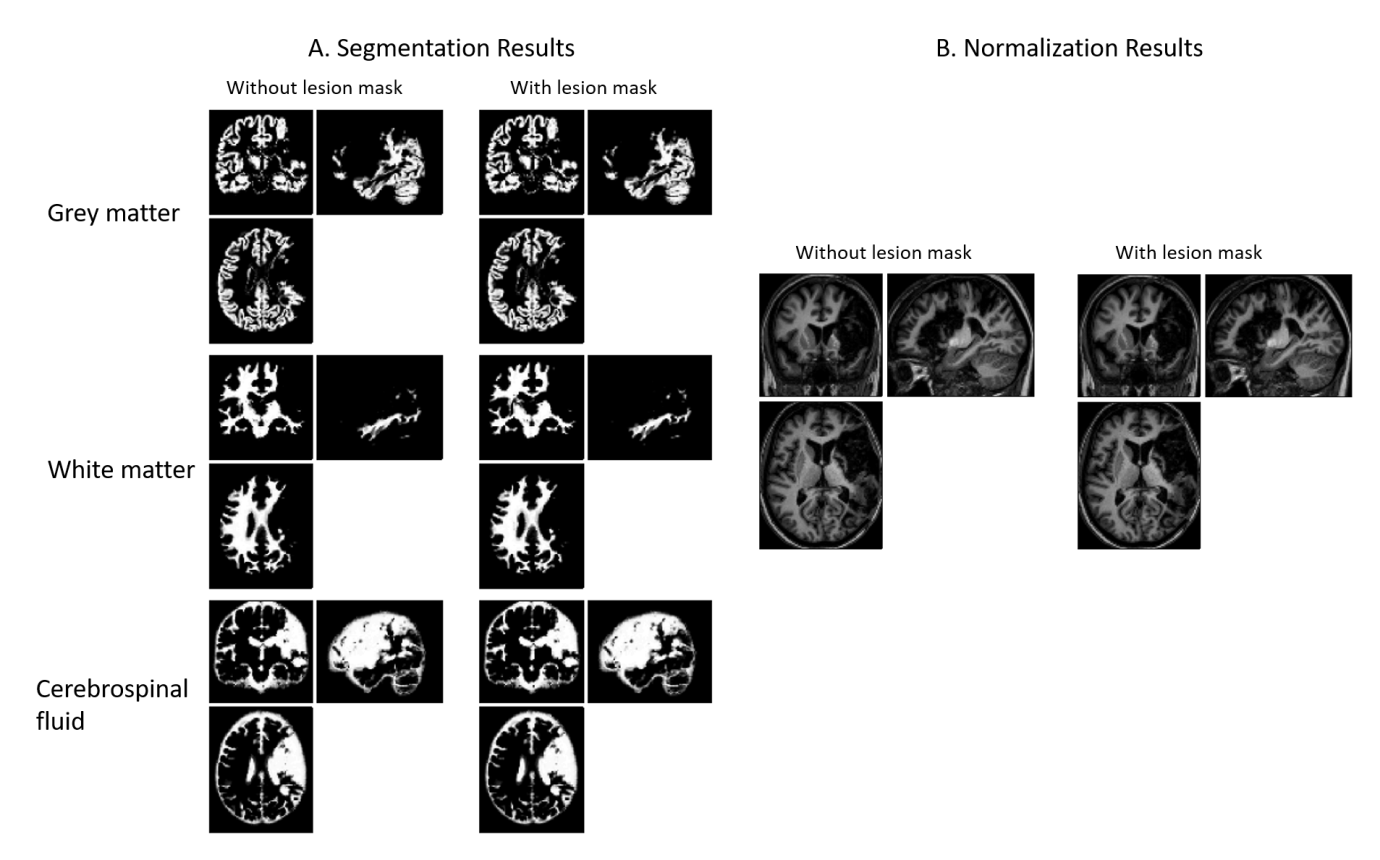


Figure S1. Segmentation (A) and normalization (B) results for a patient with a large stroke lesion after using or not the lesion mask during the preprocessing of the patient’ fMRI data. No difference is observed between the two preprocessing strategies.

The MNI coordinates and the *t* values for each activated area are reported in the supplementary tables.

Table 1. Cerebral regions showing activation in the baseline comparisons.

| Contrast / Anatomical Region | Corresponding Brodmann Area (BA) | Ipsilesional Hemisphere | | | | Contralesional Hemisphere | | | | | |
| --- | --- | --- | --- | --- | --- | --- | --- | --- | --- | --- | --- |
|  |  | x | y | z | t-value | x | y | z | t-value | | |
| **1.Passive movement of the unaffected hand (PMvt_UH)** |  |  |  |  |  |  |  |  | | |  |
| Pre/Postcentral Gyrus | 4,6,3,2 |  |  |  |  | 33 | -22 | 53 | | 11.9 | |
| Cerebellum_8 |  | -21 | -61 | -55 | 8.5 |  |  |  | | |  |
| Cerebellum_4_5 |  | -21 | -49 | -19 | 10.4 |  |  |  | | |  |
| **2.Passive movement of the affected hand**  **(PMvt_AH)** |  |  |  |  |  |  |  |  | | |  |
| Postcentral Gyrus | 2,3,5,40 |  |  |  |  | 24 | -37 | 68 | | | 7.1 |
| Insula | 13 | -33 | -22 | 11 | 6.7 |  |  |  | | |  |
| Parietal_inferior | 40,2 |  |  |  |  | 54 | -31 | 50 | | | 6.6 |
| Thalamus |  |  |  |  |  | 12 | -10 | 14 | | | 6.6 |
| Rolandic_Oper (Precentral Gyrus) | 44 | -45 | -4 | 11 | 5.6 | 48 | 2 | 8 | | | 6.5 |
| Supplemntary_Motor_Area | 6 |  |  |  |  | 9 | -7 | 65 | | | 6.5 |
| Precentral Gyrus | 4,6,3,2 | -48 | -19 | -53 | 5.5 |  |  |  | | |  |

*MNI coordinates of activated regions with their corresponding Brodmann Areas (BA), in the baseline comparisons: 1. Passive extension movement with the unaffected hand (PMvt_UH) and 2. Passive extension movement of the affected spastic hand (PMvt_AH). Significant peaks are reported at a threshold of p<0.05 corrected for Family Wise Error at the voxel level for (PMvt_UH) and at a threshold of p<0.001 uncorrected with an extent cluster volume at k=40 voxels for (PMvt_AH).*

Table 2. Cerebral regions showing activation resulting from the pairwise comparisons for the unaffected hand

| Contrast / Anatomical Region | Corresponding Brodmann Area  (BA) | Ipsilesional Hemisphere | | | | Contralesional Hemisphere | | | |
| --- | --- | --- | --- | --- | --- | --- | --- | --- | --- |
|  |  | x | y | z | t-value | x | y | z | t-value |
| **Test 1 : PM_UH > PM_UH + mT1A** |  |  |  |  |  |  |  |  |  |
| Pre/Postcentral Gyrus | 4,6,3 |  |  |  |  | 33 | -22 | 56 | 11.7 |
| Cerebellum_8 |  | -21 | -61 | -52 | 7.9 |  |  |  |  |
| Cerebellum_4_5_6 |  | -21 | -49 | -25 | 9.6 |  |  |  |  |
| **Test 2: PM_UH > PM_UH + EACov** |  |  |  |  |  |  |  |  |  |
| Pre/Postcentral Gyrus | 4,6,3,2 |  |  |  |  | 33 | -25 | 53 | 11.3 |
| Cerebellum_4_5_6 |  | -18 | -49 | -19 | 9.5 |  |  |  |  |
| Cingulum_Mid |  |  |  |  |  | 3 | -22 | 47 | 9.0 |
| **Test 3: PM_UH > PM_UH + PhysCov** |  |  |  |  |  |  |  |  |  |
|  |  |  |  |  |  | 45 | -28 | 59 | 12.4 |
| Precentral Gyrus | 4 |  |  |  |  | 39 | -16 | 59 | 10.9 |
| Postcentral Gyrus | 3 |  |  |  |  | 33 | -34 | 59 | 10.6 |
|  | 1 |  |  |  |  | 42 | -31 | 62 | 10.1 |
| Precentral Gyrus | 4 |  |  |  |  | 33 | -25 | 65 | 9.1 |
| **Test 4: PM_UH > PM_UH + EAPhysCov** |  |  |  |  |  |  |  |  |  |
| Postcentral Gyrus | 2 |  |  |  |  | 45 | -28 | 56 | 11.7 |
| Precentral Gyrus | 4 |  |  |  |  | 36 | -22 | 62 | 10.2 |
| Postcentral Gyrus | 3 |  |  |  |  | 33 | -34 | 56 | 9.4 |

*Regions in the MNI space with their corresponding Brodmann Areas (BA), showing activation resulting from the pairwise comparisons between the contrast images resulting from the different processing pathways and the baseline comparison. Test1: Effect of the addition of the lesion-masked T1 in the 1^st^ level analysis (PM_UH > PM_UH + mT1). Test2: Effect of the addition of extension amplitude regressor as covariate in the 1^st^ level analysis (PM_UH > PM_UH + EACov). Test3: The addition of physiological regressor as nuisance covariate (PM_UH > PM_UH + PhysCov). Test4: Effect of the addition of extension amplitude and physiological noise regressors as nuisance covariates (PM_UH > PM_UH + EAPhysCov). Significant peaks at a threshold of p<0.05 corrected for Family Wise Error at the voxel level are shown.*

Table 3. Cerebral regions showing activation after the pairwise comparisons for the affected hand

| Contrast / Anatomical Region | Corresponding Brodmann Area  (BA) | Ipsilesional Hemisphere | | | | Contralesional Hemisphere | | | |
| --- | --- | --- | --- | --- | --- | --- | --- | --- | --- |
|  |  | x | y | z | t-value | x | y | z | t-value |
| **Test 2 : PMvt_AH > PMvt_AH + mT1A** |  |  |  |  |  |  |  |  |  |
| Poscentral Gyrus | 2,3,5 | -45 | -19 | 53 | 5.1 | 33 | -37 | 56 | 5.2 |
|  |  | -36 | -34 | 59 | 5 |  |  |  |  |
| Cerebellum_6 |  | -27 | -55 | -28 | 4.1 |  |  |  |  |
| **Test 3: PMvt_AH > PMvt_AH + EACov** |  |  |  |  |  |  |  |  |  |
| Postcentral Gyrus | 2,3,5 |  |  |  |  | 24 | -37 | 65 | 6.5 |
| Precentral Gyrus | 6 | -27 | -16 | 59 | 5.6 |  |  |  |  |

*Regions in the MNI space with their corresponding Brodmann Areas (BA), showing activation resulting after small volume correction (SVC) applied on the pairwise comparisons between the contrast images resulting from the different processing pathways and the baseline comparison for the affected spastic hand. Test1: Effect of the addition of the lesion-masked T1 in the 1^st^ level analysis (PMvt_AH > PM_AH + mT1). Test2: Effect of the addition of extension amplitude regressor as covariate during the 1^st^ level analysis (PMvt_AH > PM_AH + EACov). Significant peaks are shown after small volume correction (SVC) applied on the activation maps, tresholded at p<0.001 uncorrected.*
